# p38 MAPK signalling regulates cytokine production in IL-33 stimulated Type 2 Innate Lymphoid cells

**DOI:** 10.1038/s41598-020-60089-0

**Published:** 2020-02-26

**Authors:** Tsvetana Petrova, Jelena Pesic, Katerina Pardali, Matthias Gaestel, J. Simon C. Arthur

**Affiliations:** 1 Division of Cell Signalling and Immunology, School of Life Sciences, Wellcome Trust Building, University of Dundee, Dundee, DD1 5EH UK; 2 Respiratory, Inflammation & Autoimmunity IMED Biotech Unit, AstraZeneca, Gothenburg, Mölndal 43183 Sweden; 3 Institute for Cell Biochemistry, Hannover Medical School, Carl‐Neuberg‐Str. 1, Hannover, 30623 Germany

**Keywords:** Stress signalling, Interleukins, Phosphorylation

## Abstract

Type 2 Innate lymphoid cells (ILC2s) are implicated in helminth infections and asthma where they play a role in the production of Th2-type cytokines. ILC2s express the IL-33 receptor and are a major cell type thought to mediate the effects of this cytokine *in vivo*. To study the signalling pathways that mediate IL-33 induced cytokine production, a culture system was set up to obtain pure populations of ILC2s from mice. Inhibitors of the p38α/β and ERK1/2 MAPK pathways reduced the production of IL-5, IL-6, IL-9, IL-13 and GM-CSF by ILC2 in response to IL-33, with inhibition of p38 having the greatest effect. MK2 and 3 are kinases activated by p38α; MK2/3 inhibitors or knockout of MK2/3 in mice reduced the production of IL-6 and IL-13 (two cytokines implicated in asthma) but not IL-5, IL-9 or GM-CSF in response to IL-33. MK2/3 inhibition also suppressed IL-6 and IL-13 production by human ILC2s. MK2/3 were required for maximal S6 phosphorylation, suggesting an input from the p38α-MK2/3 pathway to mTOR1 activation in ILC2s. The mTORC1 inhibitor rapamycin also reduced IL-6 and IL-13 production, which would be consistent with a model in which MK2/3 regulate IL-6 and IL-13 via mTORC1 activation in ILC2s.

## Introduction

Innate lymphoid cells (ILCs) are a recently identified group of lymphocytes acting within the innate immune system. ILCs lack antigen specific receptors, however in terms of the cytokines they produce they mimic various Th cell subsets. ILCs are found in both lymphoid and non-lymphoid tissues, particularly at the barrier sites including skin, respiratory and gastrointestinal systems^1–4^. Based on their effector function, cell surface markers and the transcription factors required for their development, ILCs can be subdivided into several distinct groups^1,2^. Group 1 includes NK cells and ILC1 cells, which are involved in protective immunity to viral infections and antimicrobial responses. The second group comprises of type 2 innate lymphoid cells (ILC2). ILC2 cells secrete type two cytokines and are implicated in responses to helminth infection, asthma and atopic diseases. The third group includes the lymphoid tissue inducer cells and ILC3. In 2017, a potential new group of ILC with regulatory functions was discovered. ILCreg are found in human and mouse gut and limit the activity of ILC1 and ILC3 by secreting the anti-inflammatory cytokine IL-10^5^.

The first evidence for the existence of ILC2 cells was found in 2006 when Fallon *et al*. showed that during infection with the helminth *Nippostrongylus brasilienis* a population of non-T and non-B cells secrete IL-4, IL-5 and IL-13 and promote helminth expulsion^6^. In 2010, several labs reported the characterisation of ILC2s as a distinct population of cells, which were initially referred to as either neuocytes, innate helper 2 cells or natural helper cells^7–9^. ILC2s are found in the lymphoid tissues such as spleen and mesenteric lymph nodes, as well as in some non-lymphoid organs including fat associated lymphoid clusters, lungs, skin and liver. In mice, ILC2 are characterised by the lack of expression of surface markers of other immune cells (CD3, CD4, CD8, CD19, CD11b, CD11c, F4/80, FcεR,) and the expression of the IL-7R, IL-33 receptor (ST2), IL-25 receptor (IL-17RB), KLRG1, ICOS and c-kit^10^. Human ILC2s are lineage negative and express IL-7R, the prostaglandin receptor CRTH2 and CD161^11^. ILC2 responses can be triggered by the epithelial derived cytokines IL-33, IL-25 or TSLP. In addition, lipid mediators such as prostaglandins and leukotrienes or neuronal derived neuropeptides can also induce ILC2 activation^12^.

Murine ILC2s from various tissues including mesenteric fat, lungs, bone marrow and small intestine express the IL-33 receptor chain ST2, which is encoded by the *Il1rl1* gene^13^. Human ILC2 isolated from the skin or white adipose tissues also express ST2^14^. IL-33 is considered as one of the most prominent activators of the ILC2 function^15^. IL-33 induces production of the type two cytokines both in human and murine ILC2 during *in vitro* stimulation^14,16,17^. Upon *in vivo* administration of IL-33 in mice, ILC2 are able to produce IL-5 and IL-13^18^. ILC2s are also the predominant source of IL-13 during early stage of *N. brasiliensis* infection and loss of IL-33 led to substantial reduction in the ILC2-derived IL-13 during *N. brasiliensis* without affecting the Th2 responses^8,19^. Because of their ability to mount a strong response to IL-33 stimulation, ILC2 have been proposed to be involved in the pathology of asthma^20,21^. In addition to stimulating cytokine production, IL-33 is also required for ILC2 egress from the bone marrow and as a result *Il33*^−/−^ and *Il1rl1*^−/−^ mice have an increase in ILC2 precursors in the bone marrow, but decreased numbers of mature ILC2s in the periphery^22^. This might be due to stimulation with IL-33 leading to the downregulation of the chemokine receptor CXCR4 which regulates the retention of developing leukocytes in the bone marrow^22,23^.

IL-33 is pleiotropic cytokine that plays key role in regulating both innate and adaptive immunity. IL-33 was described in 2005 by Schmitz *et al*. as a novel member of the IL-1 superfamily and that was able to bind to the orphan IL-1 Receptor family member ST2 (the suppressor of tumorigenic 2)^24^. IL-33 was later shown to be identical to a protein called nuclear factor from high endothelial venules (NF-HEV), which had previously been identified in endothelial cells^25^. IL-33 is constitutively expressed in a number of cells outside the immune system, notably epithelial and endothelial cells, where it localises to the nucleus^26–31^. Unlike IL-1β, IL-33 does not possess a secretory sequence and is not processed by the inflammasome prior to its release. Instead IL-33 is released from cells undergoing necrosis and is proposed to act as an “alarmin”^32,33^. Once released IL-33 has been proposed to act on a number of cells in addition to ILC2s including Th2 cell, Tregs and mast cells^24,34–37^. IL-33 stimulates cells via a heterodimer receptor made up of the IL-33 specific ST2 chain and the IL-1RAcP^38,39^. Like other IL-1 family receptors, the IL-33 requires the adaptor protein MyD88 to activate downstream signalling^40^. MyD88 dependent signalling has been more extensively studied in the context of IL-1 and Toll like receptor responses^41,42^, however the major upstream components of the pathway appear to be similar for IL-33 signalling. Activation of the receptor allows recruitment of MyD88, which in turn recruits IRAK4 and the IRAK1/2. Both IRAK1 and IRAK2 can interact with Traf6, resulting in the formation of hybrid K63/linear polyubiquitin chains and the activation of downstream signalling pathways including MAPK and NF-κB^43–45^. In line with this, knockout of Traf6 blocked the ability of IL-33 to activate NF-κB as well as the p38 and JNK MAPK pathways^46^. In mast cells, the p38 MAPK cascade has been found to be important for the production of a number of cytokines, including TNF, IL-6, IL-13 and GM-CSF, in response to IL-33 stimulation^47^. p38α activates the downstream kinases MK2 and MK3^42^ and in mast cells, MK2 and 3 are required for the p38α mediated regulation of TNF, IL-6, IL-13 and GM-CSF production by IL-33^47,48^.

The intracellular signalling controlling cytokines in ILC2s has not been extensively studied. One drawback to analysing signalling with ILC2s is their low number *in vivo*. We report here a method for culture of ILC2 cells from the mesenteric fat in mice. Using these cells, we show that p38 and to a lesser extent ERK1/2 are required for the IL-33 induced production of IL-5, IL-6, IL-9, IL-13 and GM-CSF in response to IL-33 treatment. Of these cytokines, IL-6 and IL-13 were regulated by the p38α activated kinases MK2 and 3, while IL-5, IL-9 and GM-CSF production were independent of MK2/3.

## Results

### ILC2s cultured from mesenteric fat respond to IL-33

**Figure 1:**
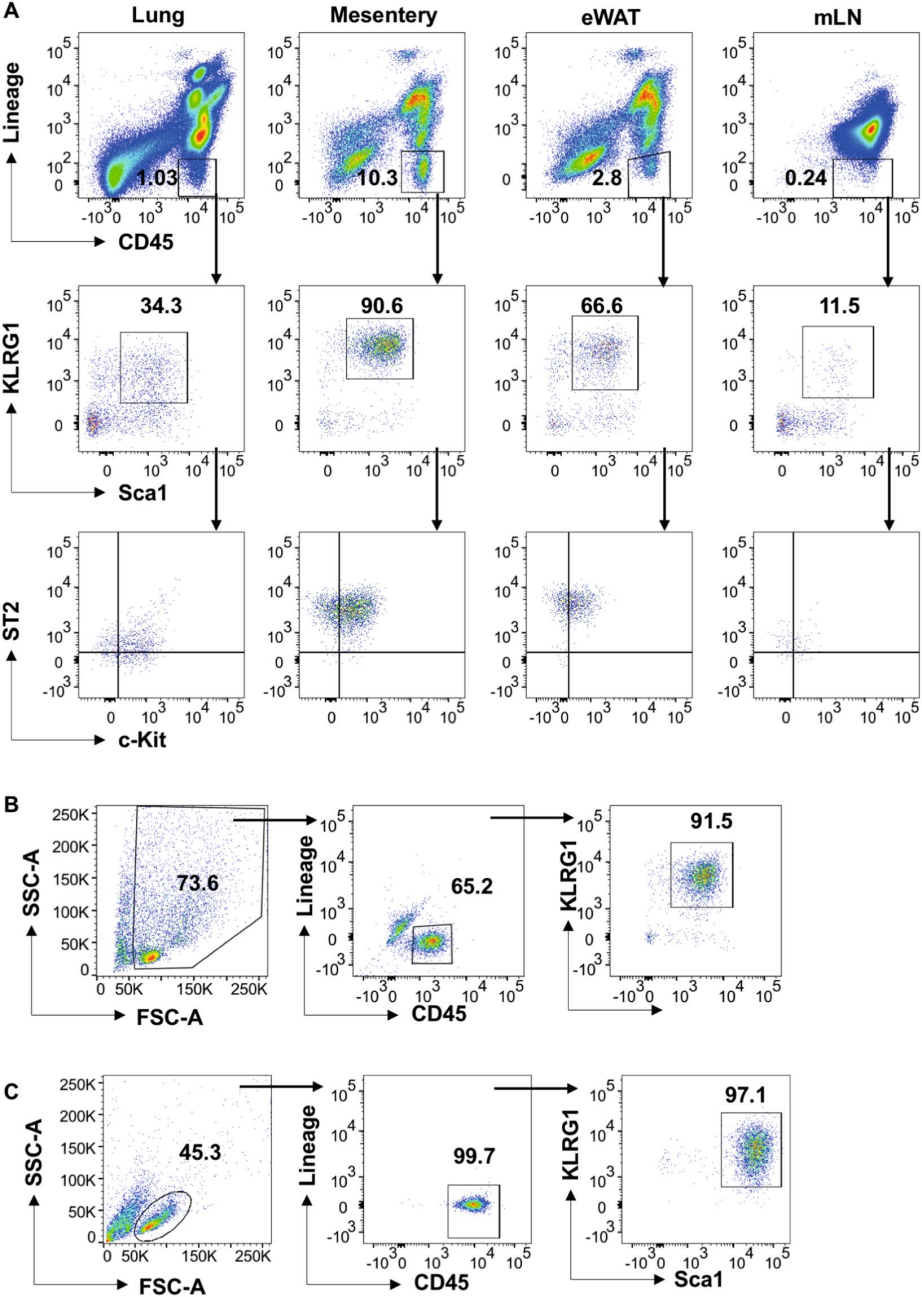
Characterisation and purification of ILC2 cells. (**A**) Flow cytometry gating of ILC2 cells in mouse lungs, mesenteric fat, epididymal white adipose tissue (WAT) and mesenteric lymph nodes (mLN). Cells were stained as described in materials and methods and doublets and were excluded using FSC-A and FSC-W. Upper panels show percentages of Lineage^−ve^CD45^+ve^ of live DAPI^−ve^ cells. The lineage negative immune cells were further analysed for expression of KLRG1 and Sca1 and ILC2 were defined as Lineage^−ve^CD45^+ve^KLRG1^+ve^Sca1^+ve^. Lower panels show expression of ST2 and c-kit in the ILC2 cells. (**B**,**C**) Representative flow cytometry plots showing the purity of ILC2 cells after depletion of Lineage positive cells (CD3ε, B220, CD19, Ly-6G/Ly-6C (Gr-1), CD11b, CD11c, TER-119, NK1.1 and F4/80) and positive selection of CD45 cells (**B**) and after magnetic sorting and culturing in IL-2 (20 ng/ml) and IL-7 (10 ng/ml) for 5 days (**C**).

In order to study the signalling pathways involved in regulating cytokine production, a culture system for ILC2s was set up. Initially ILC2 populations were analysed from the lung, white adipose tissue, mesenteric fat and lymph nodes. Single cell suspensions were prepared from these tissues and stained with CD45, a cocktail of lineage markers (CD3e, CD8, CD19, CD11b, CD11c, F4/80, Gr-1 and NK1.1), KLRG1, Sca-1 ST2 and c-kit and analysed by flow cytometry. ILC2 were defined as CD45^+ve^/Lin^−ve^/KLRG1^+ve^/Sca1^+ve^ (Fig. 1A). As a percentage of starting cells, ILC2s were present at highest levels in the mesenteric fat compared to the other tissues examined. ILC2 from the different tissues were further characterised for c-kit and ST2 expression. Of the tissues analysed, the ILC2s in the mesentery and white adipose tissue expressed the highest levels of ST2 (Fig. 1A). Based on this, the mesenteric fat was chosen as a source of ILC2s for culture to study IL-33 induced signalling.

To culture ILC2s, two rounds of magnetic sorting was used to enrich ILC2s from the mesenteric fat. The first round used a cocktail of biotinylated antibodies against CD3ε, B220, CD19, Ly-6G/Ly-6C (Gr-1), CD11b, TER-119, NK1.1 and F4/80 and streptavidin-conjugated magnetic beads to deplete other immune cells. To further enrich for ILC2 cells, a second round of magnetic selection was carried out to positively select the CD45^+ve^ cells. Flow cytometry analysis of the depleted mesenteric cells, showed that most of the other immune cells were effectively depleted and majority of the CD45^+ve^ cells were lineage negative. A population of CD45^−ve^ cells however remained following the magnetic selections (Fig. 1B). To increase the purity, the cells were then cultured in IL-7 (10 ng/mL) and IL-2 (20 ng/mL) for 5 days. At the end of this culture,>99% of the live cells were CD45^+ve^ and of these >97% were positive for the ILC2 markers Sca1 and KLRG1 (Fig. 1C). Typically a yield of 10000–20000 cells per mouse was obtained using this protocol. GATA3 is required for ILC2 development and function^49,50^. GATA3 was expressed in cultured ILC2, although levels were increased compared to freshly isolated mesenteric ILC2s. ILC2s are also reported to express receptors ST2, CD127, CD25 and c-kit^10,18^, and so the levels of these proteins on the cell surface was examined by flow cytometry. Both freshly isolated mesenteric and cultured ILC2s expressed the IL-33 receptor ST2 (Supplementary Fig. 1). Cultured ILC2s also expressed the IL-7 receptor α chain CD127 and the IL-2 receptor α chain CD25, however cell surface levels of CD127 were lower and CD25 higher than in freshly isolated ILC2s (Supplementary Fig. 1). This may reflect the use of IL-2 and IL-7 in the culture. Cultured ILC2s also expressed higher levels of c-kit than freshly activated ILC2s (Supplementary Fig. 1). This may reflect an upregulation of c-kit by cytokines in the culture media; c-kit expression on human ILC2s has been found to be upregulated by a combination of TLSP and IL-25 or TLSP and IL-33^51^. The expression of c-kit on human c-kit high and c-kit low ILC2 populations has also been found to be affected by the cytokine makeup of the culture media^52^.

### Cultured ILC2 response to IL-33

**Figure 2:**
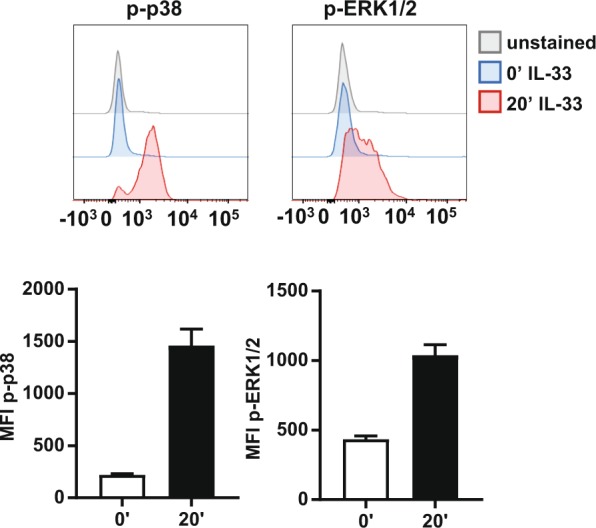
IL-33 induces activation of p38 and ERK1/2 in ILC2s. Cultured ILC2 cells were rested for 2 hours before stimulation with 100 ng/ml of IL-33 for the indicated time points. Cells were fixed, permeabilised and stained for p-ERK1/2 and p-p38. Representative histograms of p-p38 and p-ERK at 0 and 20 minutes are shown and plots show average of the median fluorescence intensity (MFI) and standard deviation (SD) of p-p38 and p-ERK1/2 from 3 independent stimulations of cultures ILC2 from one mouse. Results are representative of three experiments.

In mast cells, IL-33 has been shown to activate the p38α and ERK1/2 MAPK cascades^47^, so the activation of these pathways was used to determine if the cultured ILC2s respond to IL-33. Cells were stimulated with IL-33 and the activation of ERK1/2 and p38 determined by flow cytometry using antibodies that recognize either ERK1/2 or p38α only when phosphorylated on their TXY activation motifs. This showed that IL-33 was able to induce the activation of both ERK1/2 and p38α in the cultured ILC2s (Fig. 2).

**Figure 3:**
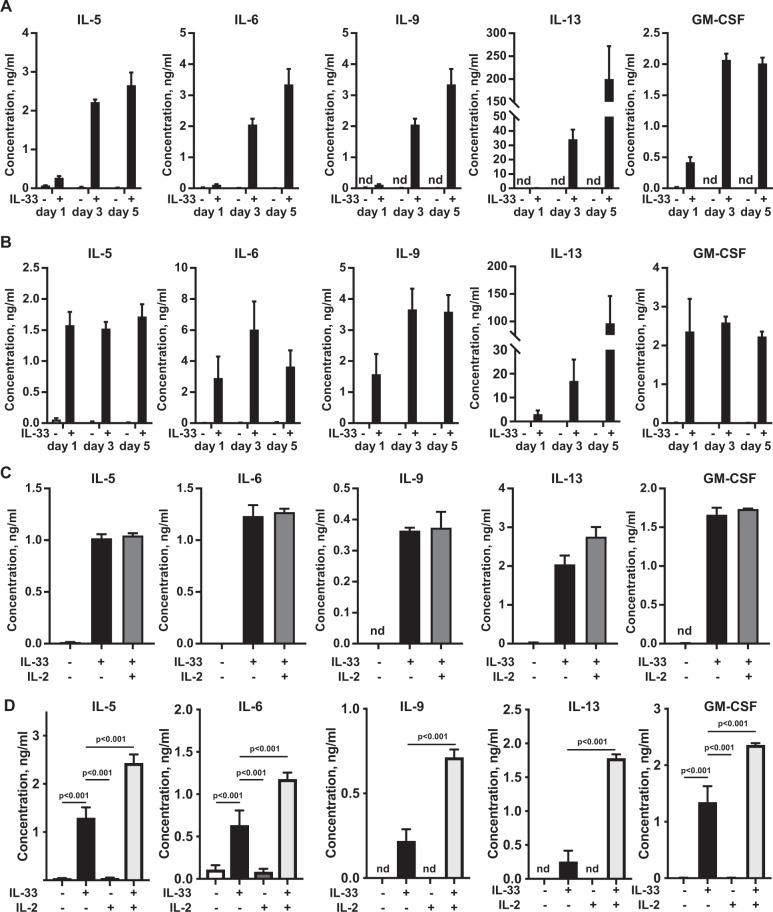
IL-33 dependent cytokine production in ILC2 cells. (**A**) ILC2 cells (DAPI^−ve^Lineage^−ve^CD45^+ve^KLRG1^+ve^Sca1^+ve^) cells were isolated from the mesenteric fat by FACS. Cells were plated at 5 × 10^3^ cells per well and stimulated *ex vivo* with IL-33 (100 ng/ml) or left unstimulated. Supernatants were collected 1, 2 and 5 days after the stimulation and IL-5, IL-6, IL-9, IL-13 and GM-CSF by multiplex cytokine assay. Plots show mean concentrations ±SD for 4 stimulations. (**B**) ILC2 cells were cultured from the mesenteric fat as described in the methods. Cells were then plated at 5 × 10^3^ cells per well with or without IL-33 (100 ng/ml). Culture media was sampled at 1, 2 and 5 days after the stimulation to measure cytokine production. Plots show mean of 4 biological replicates ±SD. (**C**) Cytokine production in cultured ILC2 cells stimulated for 24 hours with IL-33 (100 ng/ml) alone or IL-33 and IL-2 (20 ng/ml). The stimulation was done in triplicate and error bars show the mean values and standard deviation. nd indicates cytokine levels were below detectable limits in the assay. (**D**) Cultured ILC2 cells were rested for 16 h in media containing no IL-2 before stimulation with IL-33 (100 ng/ml) and IL-2 (20 ng/ml) as indicated in the figure. The stimulation was done in triplicate and error bars show the mean values and standard deviation. Significance between samples was calculated using the one-way ANOVA test followed by the Tukey’s post hoc test.

Previous reports have shown that ILC2s can produce several cytokines including IL-5, IL-6, IL-9, IL-13 and GM-CSF, and the amount of secreted cytokine present in the media increases over 3 to 5 days of stimulation. In agreement with this, ILC2s purified from the mesenteric fat by FACS, secreted IL-5, IL-6, IL-9, IL-13 and GM-CSF in response to IL-33 (Fig. 3A). While low levels of these cytokines could be detected in the culture supernatant by 24 h, their levels were increased at 3 and 5 days post stimulation. Similar to the FACS sorted ILC2s, the cultured ILC2 also produced IL-5, IL-6, IL-9, IL-13 and GM-CSF in response to IL-33, however the kinetics of cytokine production was in general faster than in the FACS sorted cells (Fig. 3B). ILC2s have also been reported to respond to IL-25 and TSLP (reviewed in^53^). Neither IL-25 or TSLP were sufficient to stimulate cytokine production on their own in the cultured ILC2s, consistent with previous reports that these cytokines on their own are not effective at inducing cytokine production in isolated ILC2s^7^. TSLP however was able to potentiate IL-33 induced production of IL-9 and IL-13 (Supplementary Fig. 2). It is possible that the presence IL-2 in the culture medium during the initial growth period having a priming effect on the ILC2s, as previous studies has shown that co-stimulation with IL-2 can increase cytokine production in response to IL-33 in ILC2s^7,21,54^. In line with this, co-stimulation with IL-33 and IL-2 did not further potentiate cytokine production in the cultured ILC2s (Fig. 3C). To further examine this, cultured ILC2s were rested in the absence of IL-2 for 16 h before stimulation with IL-33 or a combination of IL-33 and IL-2. In these experiments a combination of IL-2 and IL-33 did result in an increase in cytokine production relative to IL-33 alone (Fig. 3D).

**Figure 4:**
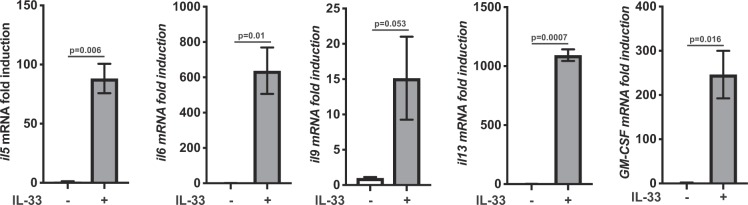
IL-33 stimulation of ILC2s increases cytokine mRNA levels. Cultured ILC2 were stimulated for 6 h with IL-33 or left unstimulated. Total RNA was then isolated and the mRNA levels for the cytokines IL-5, IL-6, IL-9, IL-13 and GM-CSF were determined by qPCR as described in the methods. Results show mean of 3 stimulations ±SD. Significance was calculated by the unpaired t-test with Welch’s correction.

In contrast to what has been observed in IL-33 stimulated mast cells, neither the *ex vivo* cells or the cultured ILC2s produced detectable levels of TNF in response to IL-33 stimulation (data not shown). In mast cells IL-33 regulates cytokine production at least in part by regulating the level of cytokine mRNAs. To determine if this also occurred in ILC2s, total RNA was isolated from control or IL-33 stimulated ILC2s and analysed by qPCR. This showed that IL-33 increased the level of the mRNA for IL-5, IL-6, IL-9, IL-13 and GM-CSF (Fig. 4).

### p38 MAPK signalling drives cytokine production in ILC2s

**Figure 5:**
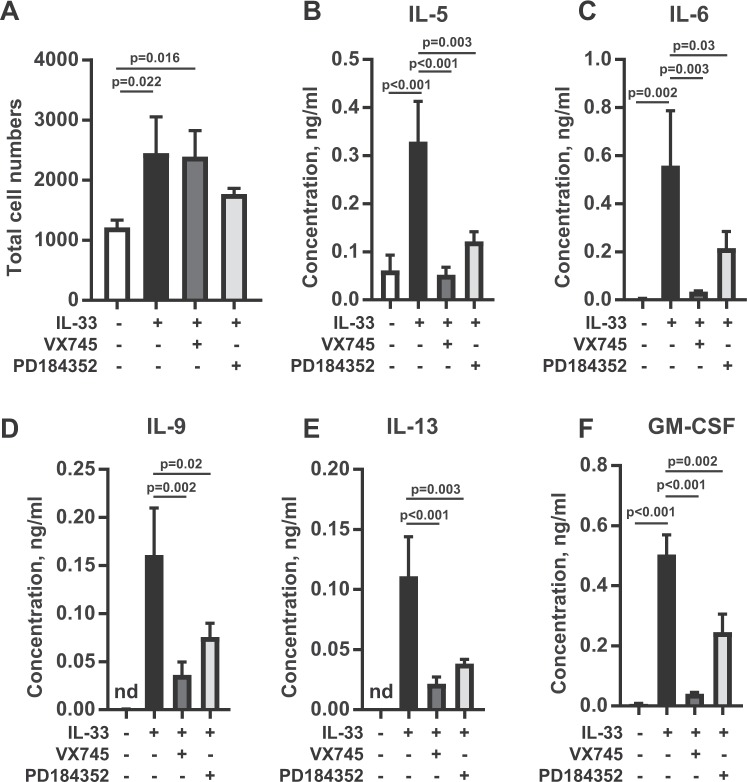
Effect of MAPK pathway inhibition on cytokine production in ILC2 cells. (**A)** Cultured ILC2 cells were plated at 5 × 10^3^ cells per well and stimulated with IL-33 (100 ng/ml) in the presence or absence of either DMSO, the p38 inhibitor VX745 (1 µM) or the MEK1/2 inhibitor PD184352 (2 µM) for 24 h. Following stimulation the cells were stained with DAPI and counted on a BD FACSVerse. Graphs show total cell numbers and represent the mean and standard deviation of 3 biological replicates. **(B**–**F)** As in A, except plots show average of IL-5, IL-6, IL-9, IL-13 and GM-CSF concentration in the culture medium at 24 h and values were normalised per 1000 cells. Significance between samples was calculated using the one-way ANOVA test followed by the Tukey’s post hoc test. N.d indicates cytokine levels were below detectable limits in the assay.

To examine the role of MAPK signalling pathways in cytokine production in ILC2 cells, specific inhibitors of the ERK1/2 and p38 MAPK pathways were used. PD184352 inhibits MKK1/2 and therefore blocks the activation of ERK1/2^55^ (Supplementary Fig. 3) while VX745 is an inhibitor of p38α and β^56,57^. Prolonged stimulation of cultured ILC2s with IL-33 for 3 to 5 days results in an increase in ILC2 number, and this was reduced by the presence of either VX745 or PD184352 (Supplementary Fig. 4). Cell cycle analysis showed that IL-33 stimulated an increase in the proportion of cells in the S and G2/M phases of the cell cycle. The addition of VX745 or PD184352 before stimulation with IL-33 did not affect the percentages of cells in the different cell cycle stages. This may indicate the inhibitors affected ILC2 survival rather than proliferation (Supplementary Fig. 5). Thus, at longer time points it is difficult to dissect an effect of the inhibitor on cell number from a direct effect of cytokine production. To avoid this complication, further experiments to study cytokine production were carried out using 24 h stimulations with IL-33 and corrected for the number of live cells at the end of the stimulation. At this time point, the presence or absence of VX745 or PD184352 did not greatly affect numbers of ILC2s in IL-33 stimulated cultures (Fig. 5A). The p38 inhibitor greatly reduced the induction of IL-5, IL-6, IL-9, IL-13 and GM-CSF (Fig. 5B–F). Blocking ERK1/2 activation with PD184352 also reduced IL-33 induced cytokine production. For IL-13, IL-9 and IL-5 the degree of inhibition approached that seen with the p38 inhibitor. For IL-6 and GM-CSF, inhibition with PD184352 was less effective than with VX745 (Fig. 5B–F).

### MK2 and 2 regulate IL-6 and IL-13 production downstream of p38 in ILC2s

As blocking p38 had the biggest effect on cytokine production, this was investigated further. In IL-33 stimulated mast cells, MK2 and 3 act downstream of p38 in the control of TNF, IL-6, IL-13 and GM-CSF production as well as the production of the chemokines CCL3 and CCL4^47^. The role of MK2 and 3 in ILC2s was therefore examined using both MK2/3 inhibitors and MK2/3 knockout mice.

**Figure 6:**
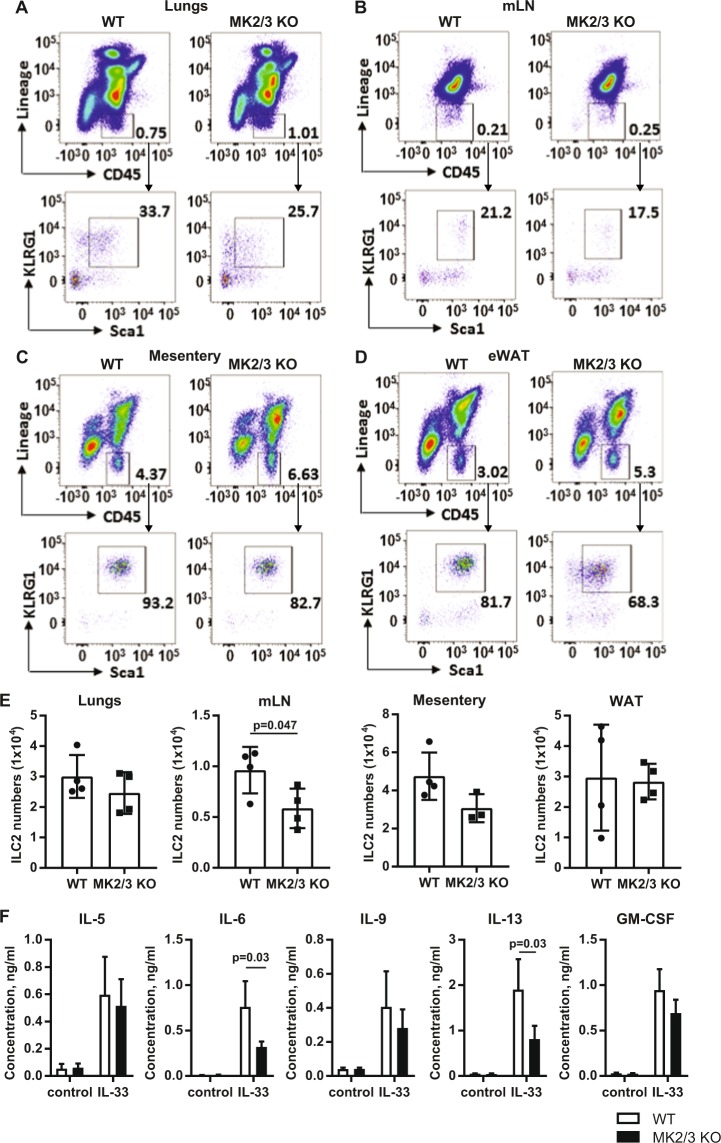
Effect of MK2/3 knockout of ILC2 numbers and IL-33 induced cytokine production. (**A**–**D)** Representative plots are showing percentage of Lineage^−ve^ CD45^+ve^ cells from all live single cells and percentage of KLRG1^+ve^ Sca1^+ve^ ILC2 cells within the Lineage^−ve^CD45^+ve^ population in lungs **(A)** mLN **(B)**, mesentery **(C)** and eWAT **(D)** of WT and MK2/3 DKO mice. **(E)** Bar plots represent mean ± SD of absolute numbers of ILC2 cells in the lungs, mLN, mesentery and eWAT for 4 mice per genotype. Symbols represent individual biological replicates. **(F)** Cultured ILC2 cells from WT and MK2/3 KO mice were stimulated with ±IL-33 (100 ng/ml) for 24 hours. Following the stimulation cells were stained with DAPI (0.5 µg/mL) and absolute counts were obtained by BD FACSVerse. Supernatants were analysed by multiplex cytokine assay. Bar plots show mean concentrations of IL-5, IL-6, IL-9, IL-13 and GM-CSF ± SD and represent the average value of 4 biological replicates (each biological replicate was made by pulling cells from 4 mice). Values were normalised per 1000 cells. Significance was calculated by the unpaired t-test with Welch’s correction **(E)** or the non-parametric Mann-Whitney test where data did not follow normal distribution **(F)**.

**Figure 7:**
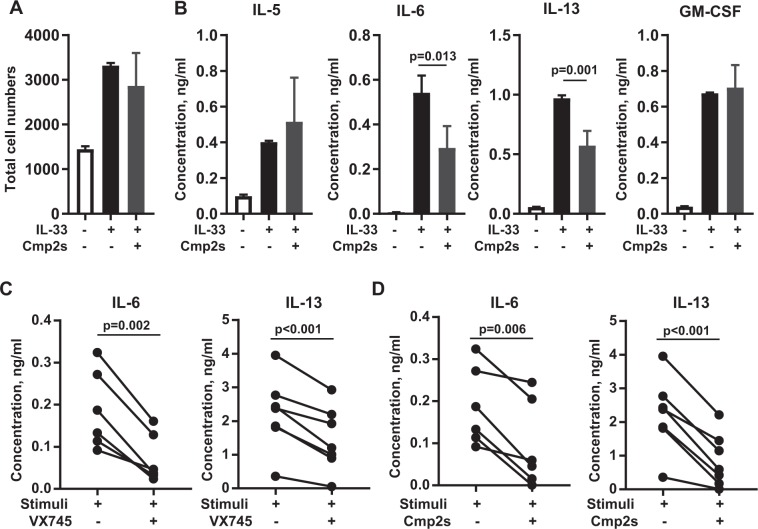
MK2/3 inhibition reduced IL-6 and IL-13 induction in ILC2s. (**A)** Total cell numbers of cultured ILC2 cells following 24 h stimulation with IL-33 in the presence or absence of Cmp2s (5 µM). **(B)** As in A, except plots show average of IL-5, IL-6, IL-13 and GM-CSF concentration in the culture medium. Values were normalised per 1000 cells. For A and B stimulations were done in triplicate and error bars show the mean value and standard deviation. **(C,D)** Human ILC2s were isolated from blood and where indicated treated with 1 µM VX745 **(C)**, 5 µM Cmp2s **(D)** or DMSO. Cells were then cultured in the presence of IL-33 (10 ng/ml), IL-2 (5 ng/ml), IL-25 (10 ng/ml) and TSLP (10 ng/ml) for 3 days. The levels of IL-6 and IL-13 present in the media were then determined as described in the methods. Graphs show comparisons between DMSO and VX745 **(C)** or DMSO and Cmp2s **(D)** for 6–7 individual donors. Statistical significance was determined using the one-way ANOVA test followed by the Tukey’s post hoc test (**B**) or paired t-test with Welch’s correction **(C,D)**.

The numbers of ILC2s in the lungs, mesenteric lymph nodes, mesenteric fat and epididymal white adipose tissue (eWAT) of MK2/3 double knockout mice were determined to check that the loss of MK2 and 3 did not affect ILC2 differentiation. Although there was an increase of the Lineage negative CD45^+ve^ immune cells in the lungs, mesentery and eWAT in the knock out mice, the percentages of KLRG1^+ve^Sca1^+ve^ ILC2 cells was lower. As a result, absolute numbers of ILC2s were not significantly different from those in the WT mice (p > 0.05, students test, Fig. 6A–E). Total numbers of ILC2 in the lymph nodes of the MK2/3 knockout mice were reduced relative to the numbers in wild type mice. Interestingly, the expression of KLRG1 was lower in the eWAT of MK2/3 knock out ILC2 (Supplementary Fig. 6). Although KLRG1 is an inhibitory ITIM motif containing receptor, the upregulation of KLRG1 expression has been associated with the activation of ILC2^58^. Hence, ILC2 in the eWAT of MK2/3 knockout mice might have a less activated phenotype. The production of IL-5, IL-9 and GM-CSF in response to IL-33 stimulation was similar between wild type and MK2/3 knockout cells, indicating that these cytokines were regulated by p38 independently of MK2 and 3. The production of IL-6 and IL-13 was however decreased in the MK2/3 knockout ILC2s relative to wild type cells (Fig. 6F). As it is possible that this could be due to a developmental role for MK2/3 rather than a direct effect of cytokine production, the effect of acutely inhibiting MK2 and 3 was examined. Cmp2s is a highly selective inhibitor of MK2 and 3. It did not affect ILC2 cell numbers following 24 h of IL-33 stimulation (Fig. 7A). Similar to the results in MK2/3 knockout cells (Fig. 7B) Cmp2s decreased the production of IL-6 and IL-13 in response to IL-33 stimulation in wild type cells. Cmp2s did not affect IL-5 or GM-CSF production. The above data indicated that a p38 – MK2/3 dependent pathway regulated IL-6 and IL-13 production in murine ILC2s. To see if something similar occurred in human ILC2s, human ILC2 cells were isolated from peripheral blood and stimulated *ex vivo*. As these cells had not been primed, they were stimulated with IL-33 in combination with IL-2, IL-25 and TSLP, a combination previously found to result in cytokine production in these cells^59^. While the absolute level of IL-6 and IL-13 produced varied across donors, pretreatment of the cells with either VX-745 or Cmp2s consistently reduced IL-6 and IL-13 production (Fig. 7C,D).

**Figure 8:**
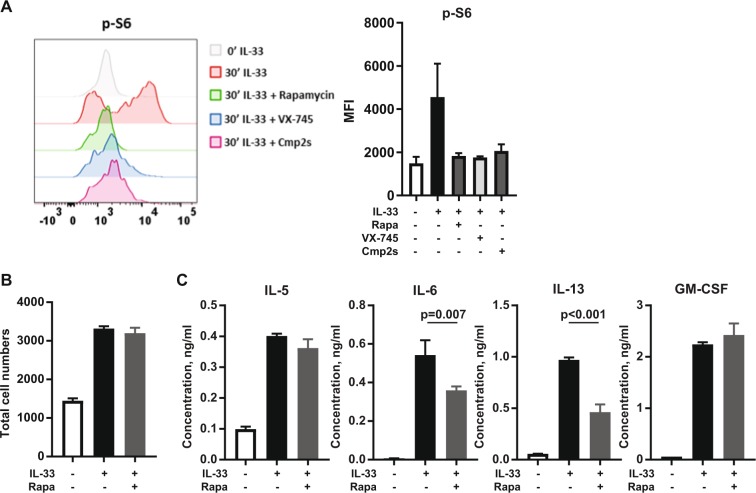
Crosstalk between MK2/3 and mTOR in ILC2 cells. (**A)** Cultured ILC2s were rested for 3 hours in ILC2 media lacking IL-2 and IL-7 and pre-incubated for a further 1 hour with 1 µM VX745, 5 µM Cmp2s or DMSO before stimulation with 100 ng/ml of IL-33 for 0 or 20 minutes. Phosphorylation of S6 ribosomal protein was measured by flow cytometry. Cells were gated based on FSC-A and SSC-A and the overlaid histograms show p-S6 (left panel). Bar plot show average of the median fluorescence intensity (MFI) of p-S6 from two biological replicates (right panel). **(B,C)** Cultured ILC2 cells were pre-incubated with DMSO or rapamycin (20 nM) for 1 hour and then stimulated with IL-33 (100 ng/mL) for 24 hours. Control cells received DMSO but not IL-33. Following the stimulation cells were stained with DAPI (0.5 µg/mL) and analysed on BD FACSVerse to determine absolute numbers of live cells. **(B)** Plot represents ILC2 absolute numbers following the stimulation. **(C)** Concentration of IL-5, IL-6, IL-13 and GM-CSF in the culture media determined by multiplex cytokine assay. (**B**,**C**) Bar plots represent the mean ± SD of stimulations done in triplicate and statistical significance was determined using the one-way ANOVA test followed by the Tukey’s post hoc test.

In CD4^+ve^ T cells, loss of MK2 and has been shown to reduce activation of the mTORC1 pathway, as judged by phosphorylation of S6 downstream of TORC1^60^, the potential for MK2/3 to regulate mTORC1 in ILC2 was examined. The treatment of ILC2s with IL-33 induced a rapid upregulation of S6 phosphorylation, which was completely reversed by treatment with the mTORC1 inhibitor rapamycin (Fig. 8A). Both the p38α/β inhibitor VX745 and the MK2 inhibitor Cmp2s blocked the IL-33 induced phosphorylation of S6 in ILC2s suggesting the p38α – MK2/3 pathway acts upstream of mTORC1 in ILC2s (Fig. 8A). To determine if this regulation might contribute to the ability of MK2/3 to regulate IL-6 and IL-13 production, ILC2 were stimulated with IL-33 in the presence and absence of the mTORC1 inhibitor rapamycin. Cell number was not affected by rapamycin following 24 h of IL-33 stimulation (Fig. 8B). The production of IL-6 and IL-13 in response to IL-33 was however reduced by the presence of rapamycin, while the levels of IL-5 and GM-CSF were unaffected (Fig. 8C).

## Discussion

We show here that inhibition of p38 blocks the production of multiple cytokines in response to IL-33. The study of cell signalling in ILC2s is restricted by the low number of these cells *in vivo* and the difficulty of obtained large numbers for study *ex vivo*. For the studies reported here, ILC2s were isolated from mesenteric fat, a tissue that contains a relatively high percentage of ILC2s, and maintained in culture using a combination of IL-2 and IL-7 (Fig. 1). Knockout mice for IL-7Ra or the common γ chain cytokine receptor lack mature ILC2s^61^ indicating an important role for IL-7 in generating ILC2s *in vivo*. Both IL-2 and IL-7 have also been reported to help maintain ILC2 survival^13,61^ while culture in combination of IL-2 and IL-7 has been used to maintain ILC2s isolated from lung or Lin^−ve^ ILC progenitors from bone marrow^62,63^. Mesenteric fat ILC2s maintained a Lin^−ve^/CD45^+ve^/Sca1^+ve^/KLRG1^+ve^ phenotype characteristic of ILC2s during culture. Similar to ILC2s directly isolated by FACS and stimulated immediately after isolation *ex vivo*, the cultured ILC2s produced IL-5, IL-6, IL-9 and IL-13 following IL-33 stimulation. The kinetics of cytokine production was faster in the cultured ILC2s than the *ex vivo* ILC2s, possibly reflecting a priming effect of IL-2 and/or IL-7; both IL-2 and IL-7 have previously been reported to have a synergistic effect with IL-33 on cytokine production in ILC2s^7,21,54^.

**Figure 9:**
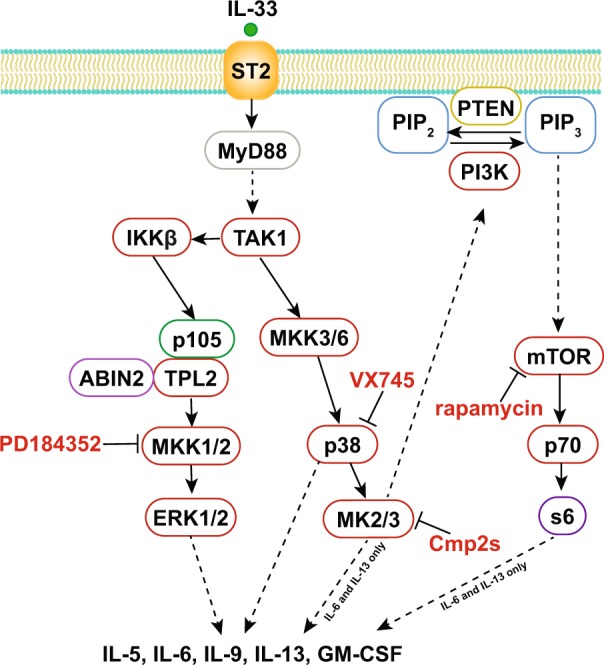
Model for MAPK regulated induction of cytokines in IL-33 stimulated ILC2s. Stimulation of ILC2s with IL-33 results in activation of the IL-33 receptor. This triggers formation of a Myddosome in which IRAK4 and then IRAK1/2 are recruited to MyD88 resulting in the activation of Traf6 and then Tak1. Tak1 activates the IKK complex, which phosphorylates p105 resulting in the activation of Tpl2, which in turn activates the ERK1/2 pathway. Tak1 also activates the p38 MAPK pathway leading to the activation of MK2 and 3. IL-33 also activates the mTOR pathway and MK2 and 3 promote activation of mTOR. The mechanism for this is unclear, but based on studies of Myd88 dependent signalling in macrophages could be due to MK2/3 promoting the generation of PIP3 at the plasma membrane. Both ERK1/2 and p38 promote the production of IL5, IL-6, IL-9, IL-13 and GM-CSF while MK2/3 and mTOR selectively drive IL-6 and IL-13 production.

We then looked at the role of MAPK signalling in regulating cytokine production in ILC2s (summarised in Fig. 9). Of the MAPK pathways tested, both the p38α/β and ERK1/2 pathways reduced the production of multiple cytokines following IL-33 stimulation, with inhibition of p38 having the biggest effect. The finding that p38α and/or β is critical for IL-33 stimulated cytokine production is in agreement with previous studies on IL-33 stimulated mast cells^47,48^, where p38 inhibition blocked IL-33 induced cytokine production. The use of VX745 does not distinguish between p38α and β, however previous studies have indicated that blocking p38α is key to the anti-inflammatory effects of p38 inhibitors both *in vivo* and in isolated macrophages^64,65^. In mast cells, MK2 and 3 were required downstream of p38α for the induction of IL-6, IL-13, GM-CSF, TNF, CCL3 and CCL4^47,48^. In contrast to mast cells, the role of MK2 and 3 in ILC2s is more restricted as blocking MK2/3 reduced IL-6 and IL-13 production in response to IL-33 but had little effect on IL-5, IL-9 and GM-CSF induction. The relative importance of MK2/3 is therefore likely to be dependent on the cell type and cytokine. In agreement with this, previous studies on other innate immune cells have also shown that the ability of MK2/3 to regulate specific cytokines is cell type specific. For example, MK2 and 3 regulate TNF production in bone marrow derived macrophages stimulated with TLR agonists but are not required for the production of IL-6 in bone marrow derived macrophages stimulated with TLR agonists^47^. In IL-33 stimulated bone marrow derived dendritic cells MK2/3 regulate IL-13 production but not that of IL-6^66^. The molecular mechanism of how MK2 and 3 regulate specific cytokines also appears to be cell type specific; for example while MK2 regulates TNF production in macrophages via the phosphorylation of TTP^67^ it regulates TNF is mast cells via a TTP independent mechanism^47^. In ILC2s one way in which MK2 and 3 may regulate IL-6 and IL-13 is via the mTORC1 pathway; p38 or MK2/3 inhibition blocked S6 phosphorylation in IL-33 stimulated ILC2s and the mTORC1 inhibitor rapamycin reduced IL-6 and IL-13 production to a similar level to that seen with MK2 inhibitors. mTORC1 regulation is complex and can be controlled by a number of inputs including PI3 kinase – Akt signalling, amino acid availability and by AMPK^68^. In macrophages the increase in PIP3 at the cell membrane seen following TLR stimulation is reduced by MK2/3 inhibition resulting in reduced Akt activation^57^. It is possible that a similar mechanism may occur in ILC2 cells, with a reduction in Akt activation resulting in reduced activation of mTORC1.

Together these results indicate that the p38α/β is essential for the induction of IL5, IL-6, IL-9, IL-13 and GM-CSF in IL-33 stimulated ILC2s. In the case of IL-6 and IL-13, this requires the activation of the downstream kinases MK2 and, which in turn feed into that activation of mTORC1. Further studies will be required to delineate the molecular mechanism by which p38 regulates IL-5, IL-9, and GM-CSF induction.

## Methods

### Animals

MK2/3 (*Mapkapk2*^tm1mgl^/*Mapkapk3*^tm1mgl^) knockout mice have been described previously^69,70^. Mice were maintained on a C57Bl/6J background and wild type mice for backcrossing obtained from Charles River Laboratories UK. Animals were maintained in specific pathogen-free conditions in line with EU and UK regulations. Non breeding mice were housed in same-sex groups, in individually ventilated sterile cages and were given standard diet R&M1 or R&M3 (SDS, Special Diets Services). Animals were maintained in rooms with controlled 12 h/12 h light/dark cycle, 21 °C temperature, and relative humidity of 45–65%. All the work was performed under a UK Home Office project license in accordance with UK and EU regulations and subject to local ethical review by the University of Dundee Ethical Review Committee.

### Isolation of murine immune cells

Digestion of lungs and mesenteric fat was performed as previously described^13^. Briefly, lungs were cut into small segments and incubated in RPMI containing 50 µg/ml Liberase TM (Sigma Aldrich, Missouri, United States) and 10 µg/ml DNaseI (Roche, Basel, Switzerland) and incubated for 45 min, at 37 °C. Tissue was passed through a 40 µm cell strainer (VWR, Pennsylvania, United States) and washed with 13 ml of 5% FBS in HBSS (Sigma Aldrich). Cell suspensions were centrifuged (450 g for 5 min at RT). Red blood cells were lysed by resuspending the pellet in 1 ml of RBC lysing Buffer (Sigma Aldrich). After 2 min at RT, cells were washed in 10 mL ice cold PBS, pelleted by centrifugation at 450 g for 5 min and resuspended in 10 ml complete RPMI media (RPMI-1640 medium with 10% heat-inactivated FBS, 50 U/ml penicillin-streptomycin, 5 mM L-glutamine, 10 mM HEPES buffer, 1 mM sodium pyruvate, 50 µM 2-mercaptoethanol) and filtered through a 40 µm cell strainer. White adipose tissue and mesenteric fat were dissected in DMEM containing 4% BSA (Sigma Aldrich). Tissues were cut into small fragments and incubated with 50 µg/ml Liberase TM (Sigma Aldrich) and 10 µg/ml DNaseI (Roche) for 60 min at 37 °C. Cells were centrifuged (450 g for 5 min at RT) and RBC were lysed as described above.

Single cell suspensions from lymph nodes were obtained by gently mashing the lymph nodes through 40 µm nylon cell strainer in 5 ml of complete RPMI media. Cells suspensions were centrifuged at 450 g for 5 minutes at room temperature (RT) and red blood cells lysed as above.

### Flow cytometry

For cell counting, cells were diluted 1:10 or 1:20 in PBS with 0.5 µg/ ml of DAPI (BioLegend, California, United States) and analysed on BD FACSVerse (BD, New Jersey, United States). Absolute numbers were calculated using FlowJo software (Tree Star, Inc., Oregon, United States). For analysis of cell surface markers, standard flow cytometry techniques were used. Briefly, cells were blocked with 50 µl rat anti-mouse CD16/CD32 Fc block (BD, New Jersey, United States) (1:50 in FACS buffer (PBS + 1% BSA(Sigma)) and incubated for 20 minutes at 4 °C. For detection of surface antigens, cells were stained with the appropriate fluorophore conjugated antibodies (listed in Table 2) for 20 minutes at 4 °C. Cells were then washed by adding 1 ml of FACS buffer and centrifuged at 450 g for 5 minutes at 4 °C. Pellets were resuspended in 400 µl of FACS buffer. For dead cell exclusion 0.5 µg/ ml of DAPI (Biolegend, California, United States) was added to the cell suspensions. For analysis of phospho-ERK1/2, phospho-p38 or phospho-S6 cells were stimulated with IL-33 for the indicated in the figures time point. Cells were then fixed by adding 4% PFA (Sigma) and incubated for 20 minutes at 4 °C. The cells were then washed twice by adding 1 ml of FACS buffer and centrifuged at 450 g for 5 minutes at 4 °C. Cells were permebalised by adding 1 ml of ice cold 90% methanol (VWR) while vortexing and then incubated overnight at −20 °C. Cells were washed by adding 1 ml of FACS, centrifuged at 450 g for 5 minutes at 4 °C and stained as described above. Samples were analysed using FACSCanto or BD LSRII Fortessa (BD) and results were further analysed by FlowJo software (Tree Star).

**Table 1:** Flow cytometry antibodies.

Target	Fluorophore	Clone	Dilution	Stock conc	Source
CD11b	FITC	M1/70	1:200	0.5 mg/ml	BioLegend
CD11c	FITC	N418	1:200	0.5 mg/ml	BioLegend
CD19	FITC	6D5	1:200	0.5 mg/ml	BioLegend
CD127	PE	A7R34	1:200	0.2 mg/ml	BioLegend
CD25	Alexaflour 700	PC61	1:200	0.5 mg/ml	BioLegend
CD3ε	FITC	17A2	1:200	0.5 mg/ml	BioLegend
CD45	APC-eFluor 780	30-F11	1:200	0.2 mg/ml	ThermoFisher
CD45	BV510	30-F11	1:200	0.2 mg/ml	BioLegend
CD8	FITC	53–6.7	1:200	0.5 mg/ml	BioLegend
c-Kit	PE/Cy7	2B8	1:200	0.2 mg/ml	BioLegend
F4/80	FITC	BM4	1:200	0.2 mg/ml	BioLegend
GATA3	PE	16E10A23	1:20	2.5 μg/ml	BioLegend
KLRG1	APC	2F1	1:200	0.2 mg/ml	BD Biosciences
Lineage	PE	mixed	1:50		BioLegend
NK1.1	FITC	PK136	1:200	0.5 mg/ml	BioLegend
Ly-6G/Ly-6C (Gr-1)	FITC	RB6-8C5	1:400	0.5 mg/ml	BioLegend
Phospho ERK1/2 (Thr202/Tyr204) Antibody	BV421	6B8B69	1:100		BioLegend
Phospho-p38 (Thr180/Tyr182)	Alexaflour647	3D7	1:100		Cell Signalling
Phospho-S6 (Ser235/236)	Alexaflour488	D57.2.2E	1:100		Cell Signalling
Sca1	APC/Cy7	D7	1:200	0.5 mg/ml	BD Biosciences
ST2	PerCP-eFluor710	RMST2-2	1:200	0.2 mg/ml	ThermoFisher
ST2	BV421	DIH9	1:200	0.2 mg/ml	BioLegend

### Culture of murine ILC2s

ILC2 cells were purified from mesenteric fat cell suspensions using magnetic sorting. 1 × 10^7^ cells were incubated in 100 µl of CD16/CD32 Fc block (BD Biosciences) diluted 1:50 in MACS buffer (PBS containing 0.5% BSA and 20 mM EDTA (Thermo Fisher Scientific, Massachusetts, United States) for 20 minutes at 4 °C. To deplete other immune cells, a cocktail of biotinylated anti-CD3, anti-CD11b, anti-CD11c, anti-Ly6C/Ly6G, anti-F4/80, anti-CD19, anti-B220, anti-NK1.1 and anti-TER119 antibodies (antibody dilutions are listed in Table 1) was added and cells were incubated for a further 20 minutes at 4 °C. Unbound antibodies were removed by washing in 10 ml of MACS buffer. Cells were incubated with streptavidin conjugated magnetic microbeads (Miltenyi Biotec, Bergisch Gladbach, Germany) for 10 min at 4 °C according to manufacturer’s instruction. After incubation, cells were washed with 10 ml MACS buffer and pelleted by centrifugation (450 g for 5 minutes). The cell pellet was then resuspended in 1 ml of MACS buffer and passed through a LD depletion column that had been pre-wetted with MACS buffer (Miltenyi Biotec) and placed on a magnetic separator. The column was washed two times by adding 1 ml of MACS buffer. The flow through and washes were collected and cells were pelleted by centrifugation (450 g for 5 min). Cells were resuspended in 100 µl of biotinylated anti-CD45 antibody diluted 1:200 in MACS buffer and incubated for 20 minutes at 4 °C. Cells were washed with MACS buffer and incubated with streptavidin microbeads for 10 minutes at 4 °C. After the incubation, the cells were washed in MACS buffer, resuspended in 1 ml of MACS buffer and passed through a pre-wetted MS column on a magnetic separator (Miltenyi Biotec). The column was washed twice with 1 ml of MACS buffer. The column was then removed from the magnetic separator and bound cells were eluted in 2 ml of MACS buffer. Cells were pelleted by centrifugation and then were cultured at a density of 0.5–1 × 10^5^ in 200 µl of complete RPMI media supplemented with IL-2 (20 ng/ml) and IL-7 (10 ng/ml) for 5 days at 37 °C and 5% CO_2._ On day 3 half of the media was replaced with fresh media containing IL-2 (20 ng/ml) and IL-7 (10 ng/ml) (PeproTech, New Jersey, United States).

### Flow cytometry sorting of murine ILC2s

Lineage negative cells were depleted using magnetic sorting as described above from mesenteric cell suspensions. Cells were then stained with anti-Lineage, anti-CD45, anti-Sca1, anti-KLRG1 and anti-ST2 (Table 2) and DAPI (0.5 µg/mL) diluted in MACS buffer. ILC2 cells were defined as DAPI^−ve^Lineage^−ve^CD45^+ve^KLRG1^+ve^Sca1^+ve^ST2^+ve^ and sorted on BD Influx™ cell sorter (BD).

**Table 2:** Biotinylated antibodies used for sorting of ILC2 cells.

Antigen	Clone	Concentration		Company
CD3ε	145–2C11	0.5 µl for 1 × 10^6^ cells	Biotin	BioLegend
CD11b	M1/70	0.5 µl for 1 × 10^6^ cells	Biotin	BioLegend
Ly-6G/Ly-6C (Gr-1)	RB6-8C5	0.5 µl for 1 × 10^6^ cells	Biotin	BioLegend
TER-119	Ter-119	0.5 µl for 1 × 10^6^ cells	Biotin	BioLegend
CD45R/B220	RA3-6B2	0.5 µl for 1 × 10^6^ cells	Biotin	BioLegend
NK 1.1	PK136	0.5 µl for 1 × 10^6^ cells	Biotin	BioLegend
CD19	6D5	0.5 µl for 1 × 10^6^ cells	Biotin	BioLegend
CD11c	N418	0.5 µl for 1 × 10^6^ cells	Biotin	BioLegend
F4/80	BM8	0.5 µl for 1 × 10^6^ cells	Biotin	BioLegend
CD45	30-F11	1:200	Biotin	BioLegend

### Cell cycle analysis

1 × 10^4^ ILC2 cells were stimulated with IL-33 (100 ng/ml) for 48 hours. Following stimulation cells were stained with Fixable Viability Dye eFluor™ 450 (eBioscience, California, United States) for 10 minutes at 4 °C according to manufacturer instructions. Cells were fixed in ice cold 70% ethanol, washed twice with PBS. Samples were then incubated for 5 min with RNase A (100 μg/ml) (Thermo Fisher Scientific), stained with Propidium iodide (50 μg/ml) (Sigma-Aldrich) and analysed using BD LSRII Fortessa (BD). Results were further analysed by FlowJo software (Tree Star).

### Stimulation of murine ILC2 cells

ILC2 cells were plated in 96 well plates at a density of 2500–5000 cells in 100 µl of complete RPMI media for stimulation. Where indicated, ILC2 cells were pre-incubated with the kinase inhibitors 1 µM VX745 (Selleckchem, #S1458), 2 µM PD184352 (Axon, #1386), 5 µM Cmp2s^47^ or 20 nM Rapamycin (Merck, #553210) for 1 hour before stimulation. Inhibitor concentrations were selected on the basis of the minimum amount required need to completely block the target kinase based on previous studies^47,56,57,71–75^. In line with results in other cells types, 2 µM PD184352 was able to completely block the phosphorylation of ERK1/2 in ILC2s (Supplementary Fig. 3). PI184352 can also target the MKK5-ERK5 pathway but this required concentrations higher than the 2 µM used in the experiments of ILC2s^75^. Inhibitors were dissolved in media containing 10% DMSO and diluted 1/100 into cell cultures, control cultures received the equivalent amount of DMSO. Cells were stimulated with 100 ng/ml IL-33 for the times indicated in the figure legends. Control cells were left unstimulated. Following the stimulation, 50 µl from the culture media was carefully collected at the times indicated without disturbing the cells to measure cytokine production. At the end of the stimulation, cells were stained by adding 150 µl of PBS with DAPI (0.5 µg/mL) in each well. Plates were analysed on a BD FACSVerse to determine absolute counts of live (DAPI negative) cells. For 5 day stimulations of ILC2, cells were plated in 100 µl of media. 50 µl of the culture media were collected on day 1 and day 3 and replaced with fresh 50 µl media with the corresponding stimuli and inhibitors. On day 5 the media was collected and cells were counted as described above.

### Human ILC2 isolation and culture

ILC2 were isolated from peripheral blood and defined as Lin^−ve^ (Lin: CD1a, CD3, CD4, CD14, CD16, CD19, CD34, CD56, CD94, CD123, CD336, CD303, FcεR1a, TCRαβ, TCRγδ)/CD127^+ve^/CD161^+ve^/CRTH2^+ve^/c-Kit^neg/pos^, as previously described^59^. ILC2 cells from healthy donors were cultured in X-vivo 15 medium (Lonza UK, BE02-060F.) containing 2% autologous human serum (Sigma-Aldrich Sweden AB, cat.Nr. H5667) at a concentration of 4 × 10^4^ cells/ml in 96-well round bottom plates (4000 cells/well), preincubated with DMSO vehicle control, 1 µM VX745 and 5 µM Cmp2s and stimulated with IL-2 (5 ng/ml), IL-25, IL-33 and TSLP (10 ng/ml each) (R&D Systems, Minneapolis, MN, United States) for 3 days at 37 °C, 5% CO_2_. Supernatants were taken for mediator analysis. The gating strategy and purity of the human ILC2s is shown in Supplementary Fig. 7.

### Cytokine assay

Supernatants from cell cultures were collected and stored in −80 prior to analysis. Cytokines in supernatants from mouse ILC2s were measured using a Luminex-based Bio-Plex assay (Bio-Rad Laboratories) using manufacturer’s instructions. Standards were reconstituted in the cell culture medium. Plates were read using a Luminex 100/200 machine and analysed using xPONENT® Software (Luminex, Texas, United States). For human ILC2s, cytokines in cell culture supernatants were measured using human U-plex kits (Meso Scale Discovery, Rockville MD, USA) according to manufacturer’s protocol.

### qPCR

For RNA isolation cultured ILC2 cells were plated at concentration 1 × 10^4^ cells per 100 μl in 96-well round bottom plates Total RNA was isolated using RNeasy Plus Micro Kit (Qiagen, Hilden, Germany) and reverse transcribed using iScript (BioRad, California, United States) according to the manufactures protocol. qPCR was carried out using Sybergreen based detection (Takara Biosciences, Kyoto, Japan). Primers sequences for IL-5 were forward: 5′-AGGCTTCCTGTCCCTACTCA-3′ and reverse: 5′-CCCCCACGGACAGTTTGATT-3′ and for IL-9 were forward 5′-ATGTTGGTGACATACATCCTTGC-3′ and reverse 5′CGGCTTTTCTGCCTTTGCATCTC-3′. Other primer sequences were as described^47^. Fold induction relative to the unstimulated control cells was calculated^76^ as described using a combination of GAPDH and 18 s RNA levels for normalization.

### Statistical analysis

Data were analysed and figures were made using GraphPad Prism 7.05 software (La Jolla, United States) and Adobe Illustrator and Indesign CC 2018 (Adobe, California, United States). The distribution was determined using the Shapiro-Wilk normality test. Pair-wise comparison of parametric and non-parametric data was done using the unpaired t-test with Welch’s correction or unpaired Mann-Whitney test, respectively. Multiple comparisons were performed using one-way ANOVA followed by Tukey’s post hoc test.

## Supplementary information

Supplementary figures.
