# Supplementary material for: p38 MAPK signalling regulates cytokine production in IL-33 stimulated Type 2 Innate Lymphoid cells
Source: Sci Rep. 2020 Feb 26;10:3479. doi: 10.1038/s41598-020-60089-0 (PMC7044202; doi:10.1038/s41598-020-60089-0)
Supplement: Supplementary file 1 — Supplementary figures. [file 41598_2020_60089_MOESM1_ESM.pdf]

p38 MAPK signalling regulates cytokine production in IL-33 stimulated Type 2 Innate Lymphoid cells.

Tsvetana Petrova[1], Jelena Pesic[2], Katerina Pardali[2], Matthias Gaestel[3] and J. Simon C. Arthur1[*]

[1] Division of Cell Signalling and Immunology, School of Life Sciences, Wellcome Trust Building, University of Dundee, Dundee, DD1 5EH, UK.

[2] Respiratory, Inflammation & Autoimmunity IMED Biotech Unit, AstraZeneca, Gothenburg, Mölndal, 43183 Sweden.

[3] Institute for Cell Biochemistry, Hannover Medical School, Carl-Neuberg-Str. 1, Hannover, 30623 Germany.

Supplementary Figure 1

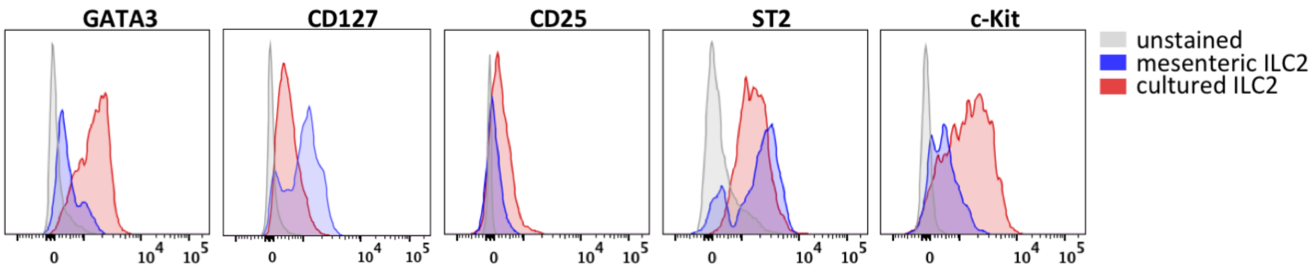

Supplementary Figure 1. Expression of GATA3, CD127, CD25, ST2 and c-Kit in "naive" mesenteric ILC2 and ILC2 cultured in IL-2 and IL-7 for 5 days.

Representative flow cytometry histograms showing expression of GATA3, CD127, CD25, ST2 and c-Kit in freshly isolated mesenteric ILC2 (blue) or ILC2 cells magnetically purified from mecentric fat and cultured in IL-2 and IL-7 for 5 days (red).

Supplementary Figure 2

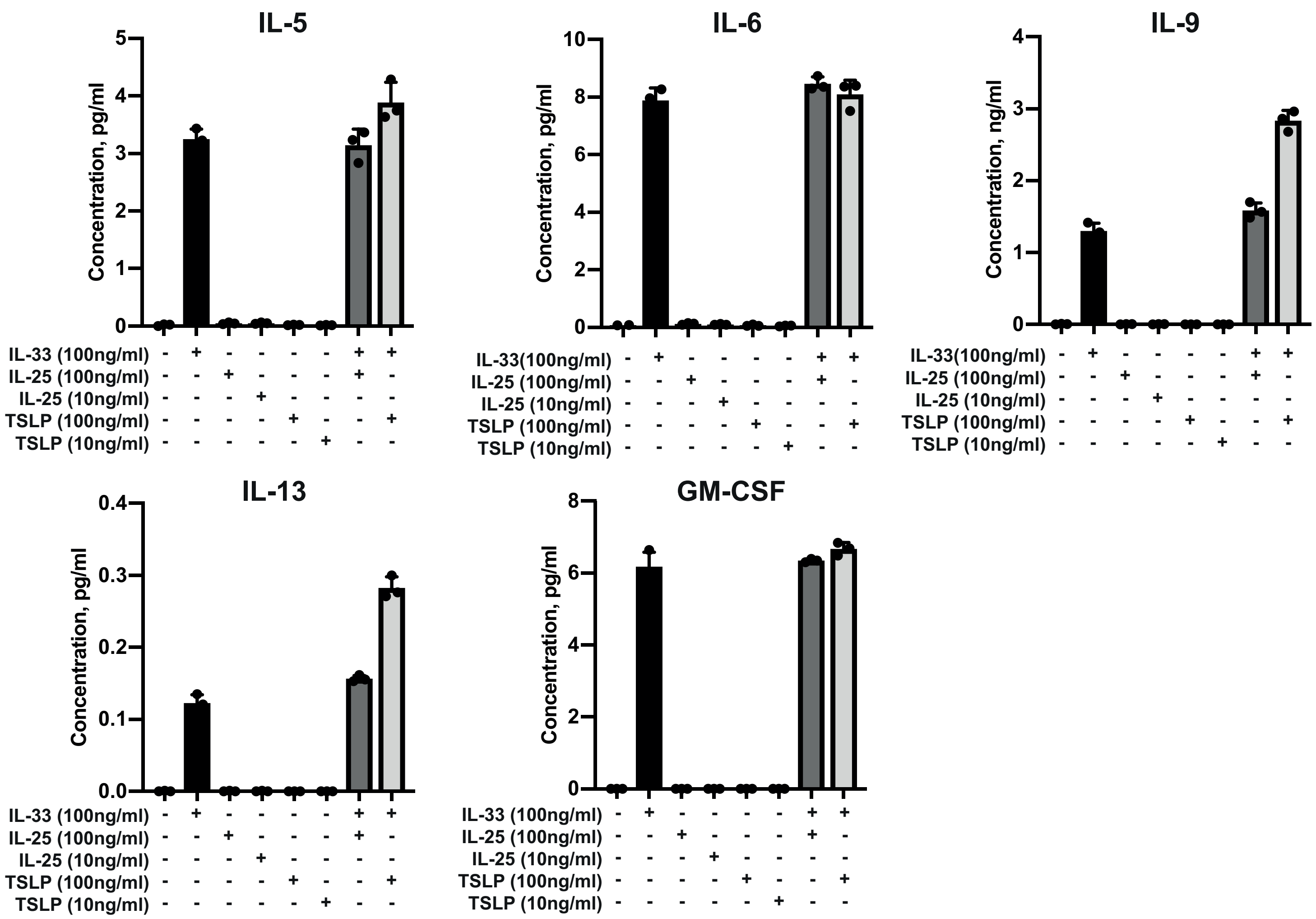

Supplementary Figure 2. IL-25 and TSLP alone do not induce cytokine production in cultured ILC2 cells.

Cultured ILC2 cells were plated at $5x10^3$ cells per well and stimulated with IL-33, IL-25 or TSLP at the indicated concentrations or left. Culture media was collected 24 hours after the stimulation and IL-5, IL-6, IL-9, IL-13 and GM-CSF concentrations were measured using multiplex cytokine assay. Stimulation was done in triplicate and bar graphs show average values +/- Standard deviation.

Supplementary Figure 3

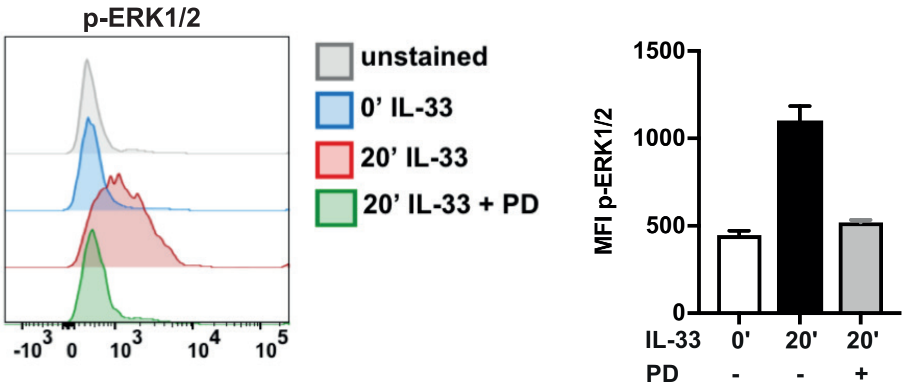

Supplementary Figure 3. MEK1/2 inhibitor PD184352 blocks phosphorylation of ERK1/2 in ILC2 cells
Cultured ILC2s were rested for 2 hours in ILC2 media lacking IL-2 and IL-7 and pre-incubated for a further 1 hour with MEK1/2 inhibitor PD184352 (2 µM) or DMSO before stimulation with 100ng/ml of IL-33 for 0 or 20 minutes. Phosphorylation of ERK1/2 was measured by flow cytometry. Cells were gated based on FSC-A and SSC-A and the overlaid histograms show p-ERK1/2 (left panel). Bar plot show average of the median fluorescence intensity (MFI) of p-ERK1/2 of 3 technical replicates. Error bars show standard deviation.

Supplementary Figure 4

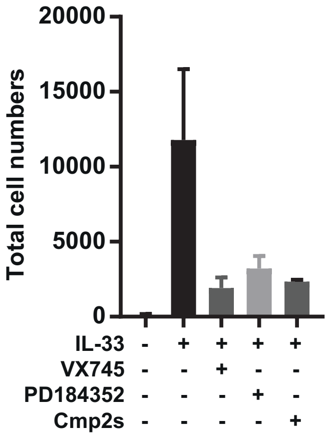

Supplementary Figure 4. Effect of MAPK pathway inhibition on ILC2 proliferation.

Cultured ILC2 cells were plated at $5x10^3$ cells per well and stimulated with IL-33 (100ng/ml) in the presence or absence of either DMSO, the p38 inhibitor VX745 (1µM), MEK1/2 inhibitor PD184352 (2 µM) and MK2/3 inhibitor Cmp2s (5µM) for 5 days. Following stimulation the cells were stained with DAPI and counted on a BD FACSVerse. Graphs show total cell numbers and represents the mean and standard deviation of 2-4 biological replicates.

Supplementary Figure 5

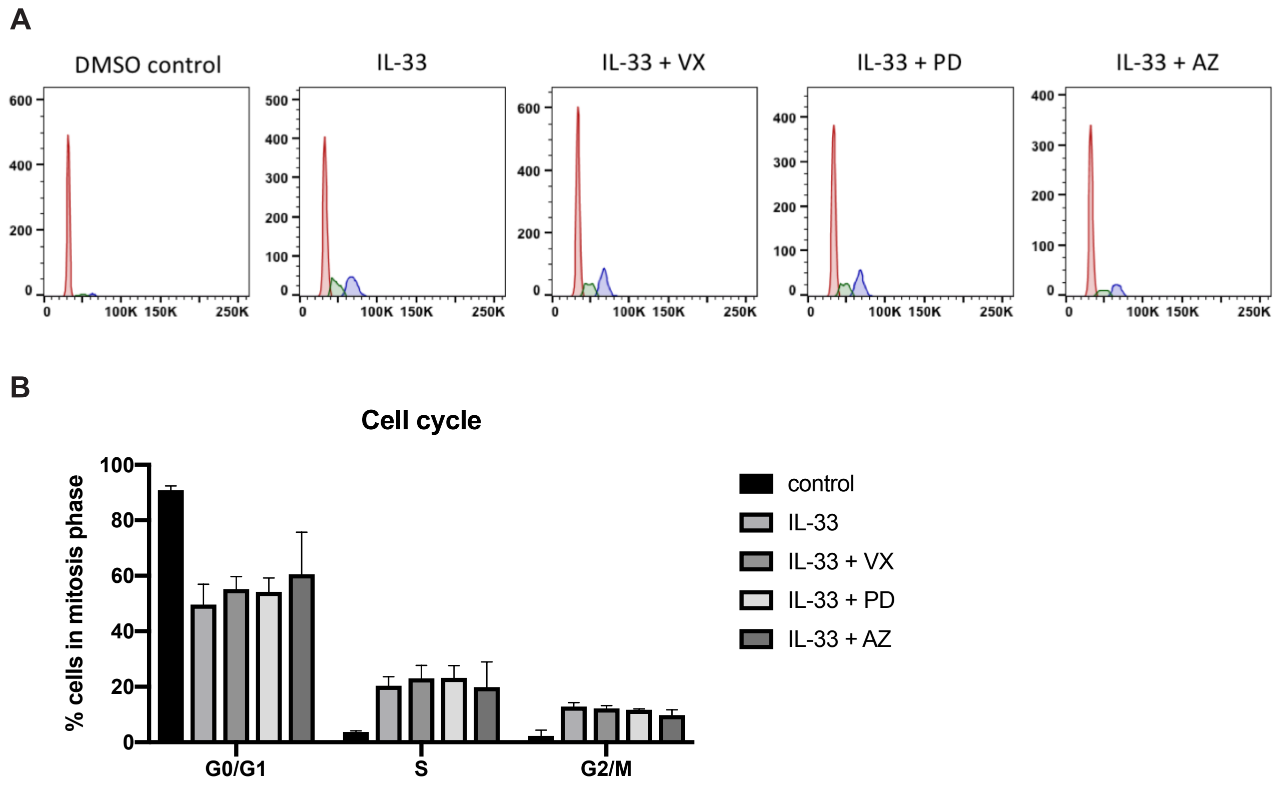

Supplementary Figure 5. Cell cycle analysis in ILC2 cells.

Cultured ILC2 cells were plated at $1x10^4$ cells per well and stimulated with IL-33 (100ng/ml) in the presence or absence of either DMSO, the p38 inhibitor VX745 (1µM), MEK1/2 inhibitor PD184352 (2 µM) and MK2/3 inhibitor Cmp2s (5µM) for 2 days. (A) Representative flow cytometry plots showing cell cycle distribution. (B) Bar graphs representing average values of 3 biological replicates. Error bars show standard deviation.

Supplementary Figure 6

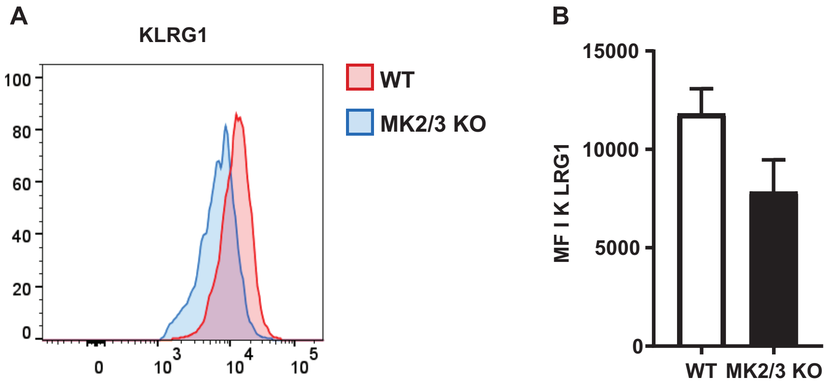

Supplementary Figure 6. KLRG1 expression in WT and MK2/3 KO ILC2 cells.

(A) Representative histogram showing the expression of KLRG1 in WT and MK2/3 KO ILC2 cells (DAPI-veLin-veKLRG1+veSca1+ve). (B) The plot shows average values of median fluorescence intensity and standard deviation of KLRG1 in WT (n=4) and MK2/3 (n=4) ILC2 cells.

Supplementary Figure 6

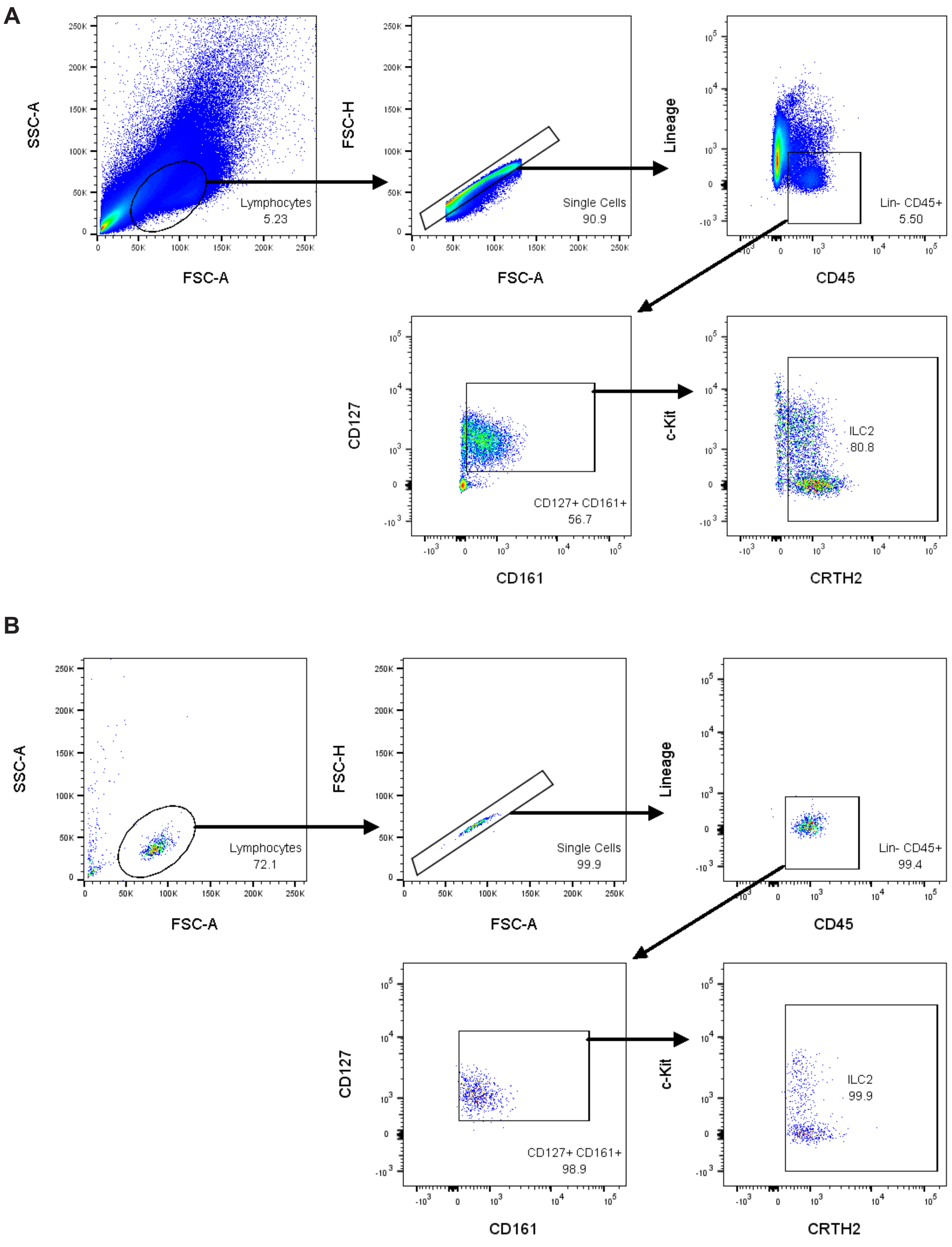

Supplementary Figure 7. Human ILC2 cells sorting strategy and purity.
(A) Representative flow cytometry plots showing gating strategy for identifying ILC2 cells in human blood. (B) Purity of ILC2 cells after sorting.
